# *Haemophilus parainfluenzae* Endocarditis Associated With Maxillary Sinusitis and Complicated by Cerebral Emboli in a Young Man

**DOI:** 10.1177/2324709617704003

**Published:** 2017-04-13

**Authors:** Anthony E. Duzenli, John Dwyer, Jeanne Carey

**Affiliations:** 1 NYU Lutheran Medical Center, Brooklyn, NY, USA

**Keywords:** *Haemophilus parainfluenzae*, infective endocarditis, HACEK group, endocarditis

## Abstract

HACEK endocarditis is often difficult to diagnose given the slow-growing characteristics of the organisms involved. *Haemophilus parainfluenzae*, one of the HACEK organisms, is an uncommon cause of endocarditis. We describe a case of a previously healthy young man with *H parainfluenzae* endocarditis that was associated with maxillary sinusitis and severe systemic complications, including septic cerebral emboli and mitral valve perforation. Previously reported cases have also described a predilection for younger people, cardiac valve pathology, and a high prevalence of stroke.

## Background

*Haemophilus parainfluenzae*, one of the HACEK organisms, is an unusual cause of endocarditis. We describe a case of *H parainfluenzae* endocarditis in which the patient’s chief complaints were persistent severe headaches and fevers. As the patient’s presentation was atypical for infective endocarditis, a primary neurologic disorder was initially suspected. Review of the literature demonstrates that previously reported cases of *H parainfluenzae* endocarditis have also been associated with severe systemic complications, including septic cerebral emboli and mitral valve perforation.^1-7^

## Case Presentation

A 27-year-old traveling musician from Canada, with a past medical history of migraines, presented to our emergency department with a 1-week history of persistent, severe headaches and intermittent fever and chills. He described the headaches as bilateral frontal headaches, which were not consistent with his typical migraines. He also reported having extreme fatigue and malaise for a few weeks prior to admission. He denied having any history of intravenous drug abuse, any recent dental work, and did not have any body piercings. He reported that he remembered being told by his mother that he was allergic to penicillin as a child but did not know the details of the nature of the allergy. He had been traveling in the United States for approximately 4 weeks. He reported that he was in a monogamous sexual relationship with a female partner.

On admission, his temperature was 97.4°F, pulse 62, blood pressure 92/59 mm Hg, and respiratory rate 18. He was alert and oriented to person, place, and time and was able to provide a history. On cardiac auscultation, a 2/6 holosystolic murmur was heard at the cardiac apex. Neurological exam was normal. Nuchal rigidity was not present. Kernig and Brudzinski signs were negative. His complaint of neck pain associated with intermittent fever and chills was initially concerning for meningitis; therefore, after obtaining a cranial computed tomography (CT) scan, a lumbar puncture was performed.

Cranial CT scan without contrast showed right-sided maxillary sinusitis. The initial cerebral spinal fluid (CSF) sample showed 13 white blood cells (WBCs), of which 80% were neutrophils; zero WBCs were present in the second tube. Ten red blood cells were present in the first tube of CSF and 8 were present in the second tube. Glucose and protein were normal at 75 and 24, respectively. Laboratory data were significant for leukocytosis of 12.3 K/UL, thrombocytopenia of 57 K/UL. Two sets of blood cultures were sent.

CSF gram stain and herpes simplex virus polymerase chain reaction were negative, and CSF culture showed no growth. HIV and influenza tests were negative. Erythrocyte sedimentation rate and C-reactive protein were both elevated at >21.2 and 31, respectively. Infectious Diseases was consulted and recommended starting broad-spectrum empiric intravenous antibiotics. As the patient had reported having a penicillin allergy, vancomycin and aztreonam were started for empiric treatment of possible bacterial meningitis. A brain magnetic resonance imaging with contrast showed a 2 × 1 cm DWI/FLAIR high signal in the left posterior temporal lobe, another 1 × 0.8 cm DWI/FLAIR high signal in the posterior corpus callosum area, and several small DWI/FLAIR high signals in the bilateral cortical surface area, which were suggestive of acute ischemic lesions.

During the first 5 days of hospitalization, the patient continued to have daily fevers with a maximum temperature of 103°F. After 3 days of incubation, blood cultures revealed gram variable bacilli from the aerobic bottles. Transthoracic echocardiogram (TTE) showed normal systolic function and mild to moderate mitral valve regurgitation. No valvular vegetations were seen. A transesophageal echocardiogram (TEE) was performed, which revealed perforation of the P2 segment of the mitral valve and an accompanying mobile mass consistent with a vegetation. Apart from the cerebral emboli, there were no other signs of systemic dissemination.

Four days into the admission, blood cultures grew pan-sensitive *H parainfluenzae*. At this point, the Infectious Diseases team narrowed the antibiotic regimen from vancomycin plus aztreonam to ceftriaxone 2 g intravenous daily, with a plan to monitor the patient closely for any adverse allergic reactions. The patient tolerated cephalosporin therapy well: his headaches lessened, and he continued to improve clinically. His platelet count normalized, his blood cultures cleared, and his fever resolved after several days of antibiotic therapy.

In summary, our patient’s diagnosis was infective endocarditis secondary to *H parainfluenzae*, complicated by septic emboli to the brain as well as mitral valve perforation. Initially, the systolic murmur heard on exam was not the focus of the diagnostic workup given that his symptoms were highly suggestive of meningitis. Mitral valve perforation and vegetation were eventually discovered via TEE. He received intravenous antibiotics before being transferred back to his home in Canada for further treatment, which would include repair of his mitral valve and completion of a 6-week course of intravenous ceftriaxone. At discharge, no apparent signs of neurological sequelae or cardiac symptoms were evident.

## Discussion

Although *H parainfluenzae* endocarditis is quite uncommon, review of the literature suggests that those who are infected by this particular type of endocarditis may share similar characteristics.^1-7^ Patients with endocarditis due to *H parainfluenzae*, and other HACEK organisms, tend to be of younger age,^1-4^ and a large majority of patients suffer from embolic phenomena, such as stroke.^1,5-7^ In addition, if antibiotic therapy is delayed, the potential for valvular damage is significant.^2,3,5,6,8^

The HACEK organisms are primarily found in the oropharyngeal flora^9^; risk factors for the development of HACEK endocarditis include dental work, nasopharyngeal infection, and even tongue piercing.^3,8^ Our patient did not complain of any oropharyngeal or upper respiratory tract symptoms; however, he was found by CT scan to have maxillary sinusitis, which has been associated with the development of *H parainfluenzae* endocarditis with cerebral emboli.^10^

Additionally, our case exhibited several relatively unique features. First, it is quite rare for endocarditis to present with headache as the primary complaint, a symptom that may have been related to the dissemination of septic emboli into meningeal vessels.^2^ Our case highlights the difficulty in diagnosing patients with endocarditis with this atypical presentation, the delay of which could lead to ineffective antibiotic administration and ultimately worse clinical outcomes. Second, *H parainfluenzae* is typically a slow-growing organism, with a mean growth time of 5 days.^2^ In our case, a high bacterial inoculum reflecting the severity of our patient’s infection may have resulted in a more rapid speciation.

Our case also emphasizes the importance of obtaining a TEE to further characterize any abnormalities detected on TTE. In our patient, while TTE showed only mitral valve regurgitation, TEE revealed mitral valve perforation and vegetation. These results are similar to the case described by Barreto et al, in which the TTE was normal and the TEE showed significant valvular perforation with vegetation.^10^ This illustrates the value of obtaining a TEE to assess for complications that may not be apparent on TTE.^11^

Furthermore, the severity of thrombocytopenia was another interesting aspect of the patient’s clinical presentation. This progressively improved with each day of antibiotic therapy. Platelets have been implicated in both the pathogenesis of, and host defense mechanism against, endocarditis.^12-14^

In conclusion, our case illustrates that *H parainfluenzae* endocarditis may present with headaches and fever, prompting an initial evaluation for a neurologic etiology. Clinicians should remain alert to the epidemiology, pathologic features, and outcome in *H parainfluenzae* endocarditis, notably the predilection for younger people, valvular damage, and the high prevalence of cerebral emboli.
